# Enhancing High-Level Food-Grade Expression of Glutamate Decarboxylase and Its Application in the Production of γ-Aminobutyric Acid

**DOI:** 10.4014/jmb.2410.10013

**Published:** 2024-12-12

**Authors:** Kang Zhang, Huihui Lv, Xinrui Yu, Xuyang Zhu, Sheng Chen, Jing Wu

**Affiliations:** 1 School of Biotechnology and Key Laboratory of Industrial Biotechnology Ministry of Education, Jiangnan University, 1800 Lihu Avenue, Wuxi 214122, P.R. China; 2 State Key Laboratory of Food Science and Resources, Jiangnan University, 1800 Lihu Avenue, Wuxi 214122, P.R. China; 3 International Joint Laboratory on Food Safety, Jiangnan University, 1800 Lihu Avenue, Wuxi 214122, P.R. China

**Keywords:** γ-Aminobutyric acid, glutamate decarboxylase, *Bacillus subtilis*, food-grade expression, whole-cell catalysis

## Abstract

Gamma-aminobutyric acid (GABA), a non-proteinogenic amino acid, exhibits diverse physiological functions and finds extensive applications in food, medicine, and various industries. Glutamate decarboxylase (GAD) can effectively convert L-glutamic acid (L-Glu) or monosodium glutamate (MSG) into GABA. However, the low food-grade expression of GAD has hindered large-scale GABA production. In this study, we aimed to elevate GAD expression in *Bacillus subtilis* through cofactor synthesis enhancement, CRISPRi-based host strain modification, and fermentation optimization. In a 3-L fermenter, the optimized strain achieved a remarkable GAD activity of 319.62 U/ml without antibiotic selection pressure, representing the highest reported food-grade expression to date. Subsequently, enzymatic property analysis facilitated the optimization of GABA production using MSG and L-Glu as substrates, achieving 100% molar conversion yields of 274.40 g/l and 481.62 g/l, respectively, with the latter yielding an unprecedented productivity of 48.16 g/l/h. Finally, *in vitro* fermentation demonstrated that GABA supplementation promoted gut microbial growth and increased the relative abundance of *Actinobacteriota* and *Bacteroidota*.

## Introduction

Gamma-aminobutyric acid (GABA), a non-proteinogenic amino acid ubiquitous in plants, animals and microorganisms [1], functions as an inhibitory neurotransmitter in the central nervous system, exhibiting numerous health benefits, such as reducing mental anxiety, lowering blood pressure, and enhancing sleep quality [2-6]. Consequently, GABA has gained widespread application in the food, pharmaceutical, and chemical industries, fueling a consistent rise in market demand [7].

**Fig. 1:**
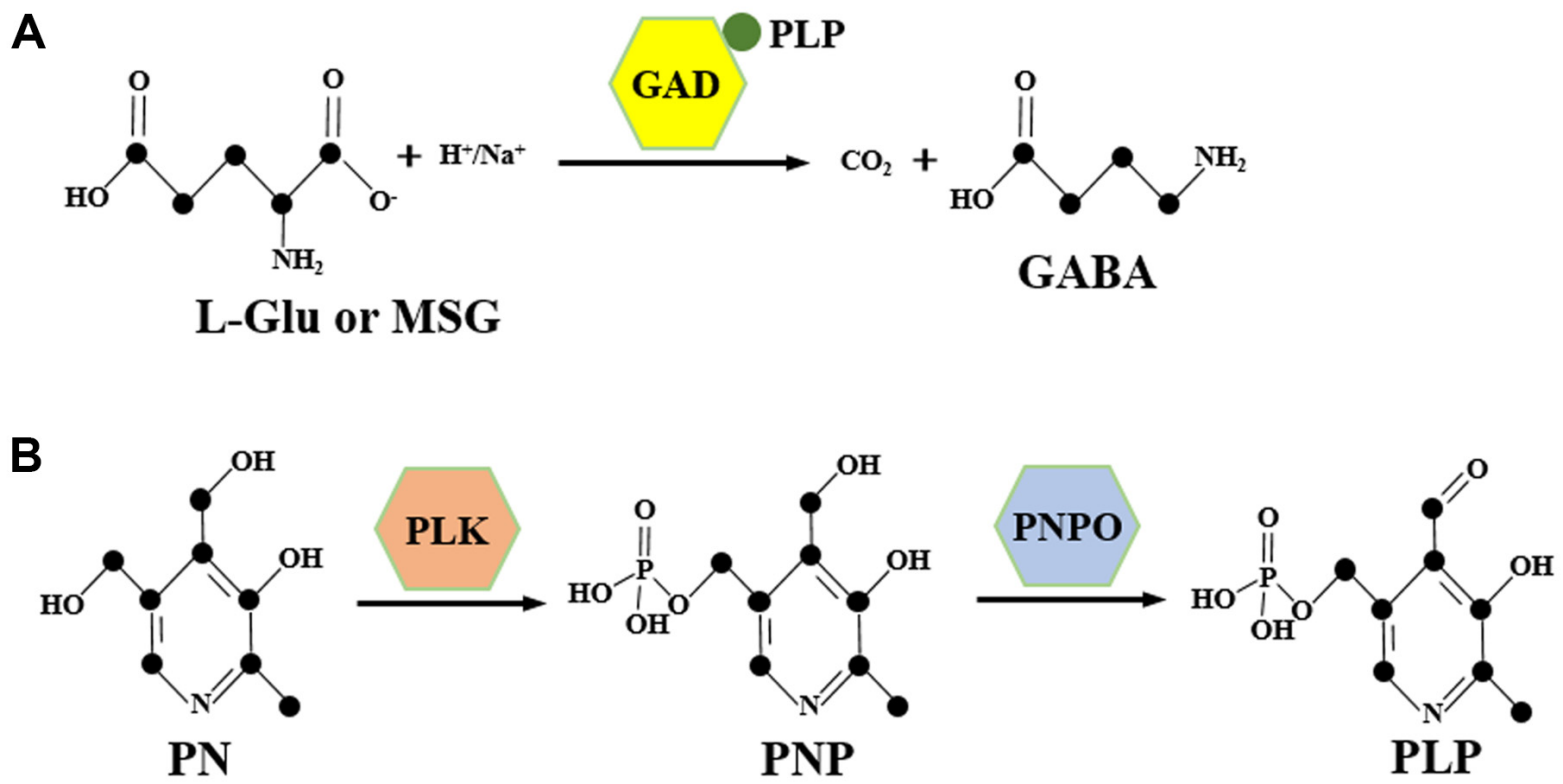
Schematic diagrams of the γ-aminobutyric acid (GABA) synthetic pathway (A) and pyridoxal 5'- phosphate (PLP) salvage pathway (B). L-Glu: L-glutamic acid; MSG: monosodium glutamate; GAD: glutamate decarboxylase; PN: pyridoxine; PNP: pyridoxine 5'-phosphate; PLK: pyridoxal kinase; PNPO: pyridoxine 5'-phosphate oxidase.

Methods for GABA production include plant enrichment, chemical synthesis, microbial fermentation, and enzyme catalysis. Among these, enzyme catalysis, encompassing free enzyme and whole-cell catalysis, directly converts L-glutamic acid (L-Glu), or monosodium glutamate (MSG), to GABA via glutamate decarboxylase (GAD), offering the advantages of simplicity, ease of purification, and high yield potential (Fig. 1A) [8, 9]. GAD is typically obtained through recombinant expression, with lactic acid bacteria commonly used due to their natural GAD activity. However, these bacteria suffer from low cell density, stringent cultivation conditions, and limited activities [10]. Although *Escherichia coli* has achieved high GAD activity through genetic engineering, safety concerns regarding endotoxins hinder its industrial application [11]. *Bacillus subtilis*, renowned for its food safety and robust secretion capabilities, has emerged as a promising host for GAD expression [12]. Currently, episomal plasmids are often utilized for GAD expression, necessitating antibiotic addition to maintain plasmid stability, which contradicts increasing health and safety standards, adds to costs, and is restricted in many countries [13].

Improving recombinant protein expression involves identifying and addressing factors limiting protein synthesis. GAD catalysis relies on the coenzyme pyridoxal 5'-phosphate (PLP), suggesting that cofactor synthesis may influence GAD expression. Promoter optimization, by modulating transcription levels, is a potent strategy to enhance exogenous protein synthesis, employed herein to improve GAD expression [14]. Additionally, during recombinant protein overexpression, imbalances in bacterial resources and energy allocation between protein synthesis and cellular metabolism can hinder expression levels. CRISPR interference (CRISPRi) technology is derived from the CRISPR/Cas system and harnesses a deactivated form of Cas9 protein known as dCas9, which features two crucial mutations (D10A and H840A) in its catalytic residues. While this modification renders dCas9 incapable of cleaving double-stranded DNA, its ability to precisely locate target sites under the guidance of single-guide RNA (sgRNA) is retained [15, 16]. Employing high-throughput screening of a CRISPRi library that encompasses a diverse mixture of sgRNAs targeting genes across the entire genome offers an efficient means to uncover crucial expression-related genes. This approach has found applications in cellular physiology and synthetic biology, and in the current study, it was utilized to enhance the expression of glutamate decarboxylase (GAD).

Building upon our previous work, in which we achieved a food-grade GAD expression system without antibiotic selection [17], we sought in this study to further elevate GAD expression through cofactor synthesis enhancement, CRISPRi-mediated host strain modification, and fermentation optimization. Enzymatic characterization of the recombinant GAD facilitated the optimization of whole-cell catalysis conditions for GABA production, ensuring efficient GABA yields. Lastly, the impact of GABA on gut microbiota was investigated using *in vitro* fermentation methods. In the current study, we report the highest-ever food-grade GAD expression and GABA productivity, and offer valuable insights for the industrial production of other amino acid derivatives.

## Materials and Methods

### Strains and Plasmids

The strains and plasmids utilized in this study are comprehensively listed in Table S1. Specifically, *B. subtilis* SCK6D served as the host for cloning and plasmid construction purposes, whereas *B. subtilis* WS9C6D was chosen as the expression host for GAD.

### Growth Media

The growth media used in this work included Luria Bertani (LB) liquid medium consisting of peptone 10.00 g/l, yeast powder 5.00 g/l, and NaCl 10.00 g/l. Next, an LB solid medium was composed of peptone 10.00 g/l, yeast powder 5.00 g/l, NaCl 10.00 g/l, and agar powder 15-20 g/l. The Terrific Broth (TB) medium contained glycerol 5.00 g/l, tryptone 12.00 g/l, yeast extract 24.00 g/l, K_2_HPO_4_·3H_2_O 16.43 g/l, and KH_2_PO_4_ 2.31 g/l. Also employed was a 3-L fermenter basic medium that consisted of glycerol 5.00 g/l, porcine bone peptone 28.00 g/l, Na_2_SO_3_ 2.00 g/l, (NH_4_)_2_SO_4_ 2.68 g/l, K_2_HPO_4_ 4.00 g/l, NaH_2_PO_4_ 4.00 g/l, MgSO_4_·7H_2_O 1.00 g/l, ammonium citrate 1.00 g/l, and trace element solution 3.00 ml/l [18]. In addition, the 3-L fermenter feed medium was composed of glycerol 300.00 g/l, MgSO_4_ 7H_2_O 7.89 g/l, porcine bone peptone 63.36 g/l, and trace element solution 40.00 ml/l. Finally, the gut microbiota culture medium contained tryptone 2.00 g/l, yeast extract 2.00 g/l, K_2_HPO_4_ 0.04 g/l, KH_2_PO_4_ 0.04 g/l, NaCl 0.10 g/l, CaCl_2_ 2H_2_O 0.01 g/l, MgSO_4_·7H_2_O 0.01 g/l, NaHCO_3_ 0.50 g/l, cysteine salt 0.50 g/l, bile salt 0.50 g/l, and Tween 80 2.00 g/l.

### Cultivation Conditions

For shake-flask cultivation, conventional construction strains and GAD recombinant strains stored at -80°C were inoculated into LB liquid medium at a 0.2% (v/v) inoculation rate, followed by incubation at 37°C and 200 rpm for 10-12 h. For GAD expression, the bacterial culture was subsequently transferred to TB medium at a 5% (v/v) inoculation rate and cultivated at 37°C and 200 rpm for 2 h. Subsequently, pyridoxine (PN) was added to a final concentration of 0.2 mM, and fermentation was carried out at 33°C and 200 rpm for 36 h.

For 3-L fermenter cultivation, the GAD recombinant strain was first streaked on an LB plate and cultivated at 37°C for 10-12 h for activation. A single colony was then inoculated into LB liquid medium and cultivated at 37°C for 10 h. This pre-culture was transferred to two bottles containing 50 ml of LB liquid medium, each with a 0.2%(v/v) inoculation rate, and cultivated at 37°C for another 10 h. The resulting bacterial solution was inoculated into a 3-L fermenter containing 0.9 L of basic medium for high-density cultivation. PN was added initially to a final concentration of 3 mM and supplemented every 24 h. The fermentation process was conducted at 37°C, with pH maintained at 6.8 and dissolved oxygen at 30%. The dissolved oxygen was adjusted through a combination of stirring shaft rotation speed and pure oxygen flow rate. When the rotation speed decreased and dissolved oxygen increased abruptly, indicating exhaustion of the primary carbon source, feed medium was introduced into the fermenter at a flow rate gradually increased from 0.02 to 0.1 ml/min. During fermentation, cell density (OD_600_) and GAD activity were regularly measured.

### Construction of Recombinant Strains Containing Different Promoters

The primers used in this study were listed in Table S2. For promoter P*_HpaII_*-P*_amyQ'_* optimization, the plasmid pUBDAL-*gadA* served as the template for PCR amplification of the vector fragment using primer pair P1/P2. The genome of strain WS9C6D was utilized as the template for PCR amplification of promoter fragments P*_spoVG_*, P*_ahpF_*, P*_tufA_* and P*_ylb’_*, employing primer pairs P3/P4, P5/P6, P7/P8, and P9/P10, respectively. Similarly, for promoter P*_aprE_* optimization, the plasmid pUBDAL-*gadA* was used as the template for vector fragment amplification with primer pair P11/P12. The plasmid pAD123-P_43_-CK was employed as the template for PCR amplification of the promoter fragment P_43_, with primer pair P13/P14. The genome of WS9C6D served as the template for PCR amplification of promoter fragments P*_spoVG_*, P*_tufA_*, P*_nprE_*, P*_ahpF_* and P*_fusA_*, utilizing primer pairs P15/P16, P17/P18, P19/P20, P21/P22, and P23/P24, respectively. These vector and promoter fragments were then ligated following the POE-PCR method, and the resulting PCR products were transformed into *B. subtilis* SCK6D to construct recombinant plasmids harboring distinct promoters [19]. The verified recombinant plasmids were subsequently transformed into the expression host WS9C6D, yielding recombinant strains with GAD or pyridoxine 5'-phosphate oxidase (PNPO) promoter substitutions.

### Construction of Recombinant Strain for High-Throughput Screening

Plasmids pUBDAL-*gadA* and pUB110-GFP11-GFP1-10 were utilized as templates for PCR amplification of the vector fragment and Split-GFP expression fragment, respectively, using primer pairs P25/P26 and P27/P28. The amplified fragments were ligated via the POE-PCR method, yielding plasmid pUB110-*gadA*-GFP11-GFP1-10. This plasmid was then transformed into the screening strain WS9Cd, which had previously integrated the *dcas9* expression cassette at the *amyE* site of the genome, resulting in recombinant strain WS9Cd-GAD-GFP.

### CRISPRi Screening and Gene Knockout

The sgRNA mixture library synthesized on the pAD123 vector was transferred into recombinant strain WS9Cd-GAD-GFP and incubated overnight on an LB plate containing 5 μg/ml chloramphenicol and 30 μg/ml kanamycin. The empty pAD123-P_43_-CK vector, lacking N20 sequence on sgRNA, served as a control. Single colonies were selected and transferred to a 96-well, shallow-well plate containing 140 μl of LB liquid medium, which was then incubated at 37°C and 750 rpm for 10 h. Subsequently, 40 μl of the seed solution was transferred to a 96-well, deep-well plate containing 600 μl of TB medium supplemented with 0.2 mM PN. After incubation at 37°C and 900 rpm for 2 h, the culture temperature was lowered to 33°C for 36 h of cultivation. Following fermentation, the culture solution was centrifuged at 4,000 ×*g* for 20 min. The cells were then washed twice with PBS buffer (NaCl 8.00 g/l, Na_2_HPO_4_ 3.58 g/l, KCl 0.20 g/l, and KH_2_PO_4_ 0.27 g/l) and resuspended in 600 μl of PBS buffer. The fluorescence of the suspended solution was measured at an excitation/emission wavelength of 488/530 nm. Strains exhibiting high fluorescence were selected for shake-flask cultivation, and those with elevated GAD expression were chosen for DNA sequencing and identification.

The screened *yqhH* gene was knocked out as follows: the genome of strain WS9C6D served as the template for PCR amplification of the upstream and downstream fragments of the homologous repair using primer pairs P29/P30 and P31/P32, respectively. Plasmid pHY300PLK served as the template for PCR amplification of lox72-Tet-lox66 with primer pair P33/P34. These three fragments were ligated via the POE-PCR method and transformed into WS9C6D, with transformants screened using 20 mg/l (w/v) tetracycline to obtain a knockout strain that integrated the Tet resistance gene. This knockout strain was then transformed with plasmid pE194-Cre and induced with 1 mM IPTG for Cre expression, resulting in a Tet-eliminating strain. Plasmid pE194-Cre was eliminated by culturing at 51°C for 12 h, yielding strain WS9C6DY, which was transformed with a recombinant plasmid for GAD expression.

### Enzyme Activity Determination and Protein Purification

A bacterial solution with an OD_600_ of 5.00 was centrifuged and collected. The cells were resuspended in phosphate-citrate buffer (pH 4.5, 50 mM) containing 0.15 mM PLP, lysed by ultrasound, and centrifuged to obtain the intracellular supernatant containing GAD protein. A substrate solution was prepared by dissolving 0.1 M MSG and 0.15 mM PLP in 50 mM of pH 4.5 phosphate-citrate buffer and preheating it at 37°C for 10 min. Subsequently, 40 μl of enzyme solution was added to 360 μl of substrate solution and reacted for 10 min. The reaction was terminated by adding 600 μl of boric acid buffer (pH 10.0) and treating the mixture in a boiling water bath for 10 min. The amounts of reaction product and substrate were determined using the HPLC-OPA method [20].

Purification was performed using the AKTA avant protein purification system and a HiTrap SP HP cation purification column (GE, USA). A UV detector set at 280 nm was used to monitor purified proteins, and the collected protein solutions were analyzed by SDS-PAGE. Protein concentration was measured using the Bradford protein concentration assay kit (Beyotime Biotechnology, China).

### Preparation of GABA by Whole-Cell Catalysis

The fermentation solution of GAD recombinant strain was centrifuged at 4°C and 8,000 ×*g* for 15 min, with the supernatant removed and the bacterial pellet collected. The pellet was washed twice with ddH_2_O and then resuspended in an appropriate amount of ddH_2_O. A substrate solution was prepared at a specific concentration using 50 mM of pH 4.5 phosphate-citrate buffer and placed in a 150 ml stoppered conical flask, to which a certain volume of GAD-recombinant bacterial cell solution was added based on enzyme activity and substrate concentration, with an initial reaction volume of 20 ml. The catalysis system was incubated in a water bath shaker and the pH was adjusted with 6 mM H_2_SO_4_ during the reaction. After completion, the GABA content was detected using the HPLC-OPA method, and the molar conversion rate was calculated.

### *In Vitro* Fermentation of Gut Microbiota

Fecal mixed bacterial samples were donated by three healthy volunteers [21]. A total of 5 ml of the fecal mixed bacterial solution was inoculated into a gut microbiota culture medium containing 5 g/l GABA, with a fermentation volume of 50 ml. A sample without GABA was used as a control. The cultivation occurred in an anaerobic workstation (80% N2, 10% CO_2_, and 10% H_2_) at 37°C for 48 h. Samples were taken every 12 h to monitor bacterial growth, pH, and organic acid content [21]. The bacterial fermentation solution was then centrifuged at 4°C and 8,000 ×*g* for 10 min, and the collected bacterial cells were used for microbial component analysis. The genome of collected cells was extracted and used as a template for PCR amplification of the V3 and V4 regions of 16S rDNA using primer pair P35/P36. The amplified products were ligated with adapters and subjected to high-throughput sequencing on the Illumina platform (Genedenovo Biotechnology, China). The microbial community structure was analyzed on the Omicsmart platform (http://www.omicsmart.com) using cluster analysis to classify operational taxonomic units (OTUs) with 97% similarity. The Shannon diversity index plot was utilized to assess microbial α-diversity, reflecting the richness and diversity of the microbial communities. Principal coordinate analysis (PCoA) and the Bray-Curtis algorithm were used for sample grouping analysis.

### SDS-PAGE Analysis

Bacterial cell lysates or GAD purification samples were centrifuged at 12,000 ×*g* for 5 min, and 20 μl of the supernatant was mixed with 5 μl of 5x protein loading buffer. The mixture was incubated in a boiling water bath for 10 min and then centrifuged at 12,000 ×*g* for 30 s. Eight μl of the supernatant was loaded onto a protein gel for electrophoresis, conducted at 120 V. The gel was then stained with Coomassie Brilliant Blue R250.

### Data Analysis

All data were derived from at least three independent experiments, and results were expressed as mean ± SD. Statistical analysis was conducted using Student’s *t*-test, with results considered significant at *p* < 0.05.

## Results and Discussion

### Enhancing GAD Activity Based on Promoter Optimization

GAD is an enzyme that requires high-cost coenzyme PLP. The endogenous synthesis level of PLP in bacteria is often insufficient to meet the demands of overexpressed GAD [22]. Most microorganisms can form a PLP salvage pathway through the sequential catalytic reactions of pyridoxal kinase (PLK) and PNPO, whereby the low-cost substrate PN is converted to pyridoxine 5'-phosphate (PNP) by PLK, and subsequently to coenzyme PLP by PNPO (Fig. 1B) [23]. In *B. subtilis*, the absence of PNPO hinders the conversion of PN to PLP. In our previous study, we successfully constructed a synthesis pathway for PLP from PN in *B. subtilis* by co-expressing *E. coli* PNPO [24]. In this study, the transcription levels of GAD- and PNPO-encoding genes were regulated by optimizing their promoters to achieve higher GAD activity.

**Fig. 2:**
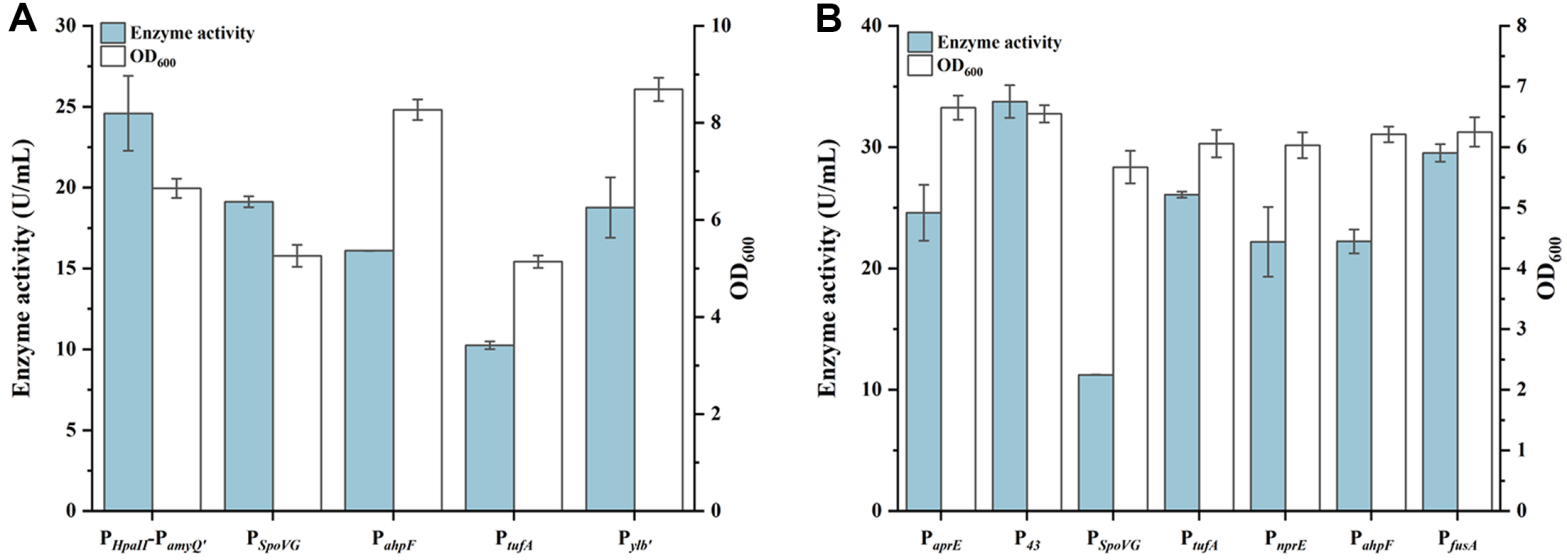
Enhancement of GAD activity through promoter optimization. (**A**) GAD promoter optimization. (**B**) PNPO promoter optimization.

The recombinant strain WS9C6D-GAD, which integrates seven copies of the GAD expression cassette into its genome and harbors the episomal plasmid pUBDAL-*gadA* with the auxotrophic selection marker *dal*, was constructed in our previous study [17]. This strain can express GAD without the need for antibiotic screening pressure and served as the starting strain for modifications aimed at enhancing GAD activity. Given the high copy number of the episomal plasmid, the transcription level of GAD-encoding gene was regulated by replacing the original promoter P*_HpaII_*-P*_amyQ'_* in pUBDAL-*gadA* with alternative promoters P*_spoVG_*, P*_ahpF_*, P*_tufA_* and Pylb', respectively (Table 1). The GAD enzyme activities of the recombinant strains featuring these different promoters were measured after 36 h of shake-flask fermentation. As shown in Fig. 2A, the strain WS9C6D-GAD with the original promoter P*_HpaII_*-P*_amyQ'_* exhibited the highest enzyme activity of 24.81 U/ml, while the other promoters showed no positive effects. Subsequently, the original promoter P*_aprE_* of PNPO in pUBDAL-*gadA* was replaced with P_43_, P*_spoVG_*, P*_tufA_*, P*_nprE_*, P*_ahpF_*, and P*_fusA_*, respectively (Table 1). As shown in Fig. 2B, after 36 h of shake-flask fermentation, the recombinant strain WS9C6D-GAD-P_43_ with promoter P_43_ exhibited the highest GAD activity of 33.75 U/ml, which was 1.37-fold that of the control strain WS9C6D-GAD. Promoter P_43_, derived from *B. subtilis*, is a strong constitutive promoter that mediates transcription of cytidine dehydrogenase-encoding gene [25]. Replacing the original promoter P*_aprE_* with P_43_ significantly increased GAD activity, likely due to the enhanced expression level of PNPO. This higher PNPO expression promotes the conversion of PN to PLP, resulting in increased coenzyme availability, and consequently, enhanced GAD activity.

### Enhancing GAD Activity Using CRISPRi Screening Technology

To further enhance GAD activity, CRISPRi screening technology was employed to identify crucial factors limiting GAD expression, and a high-throughput screening system was constructed based on Split-GFP. In our previous work, a CRISPRi screening system targeting 4,237 coding sequences in the *B. subtilis* 168 genome was developed, achieving a coverage rate of 99.7% with 6,000 sgRNAs [26]. This system comprises two main components: the *dcas9* expression cassette, driven by the xylose-inducible promoter P*_xylA_* and inserted at the *amyE* site in the genome, and the sgRNA expression cassette, driven by the constitutive promoter P_43_ and located on the plasmid pAD123. This CRISPRi screening system was utilized in this study.

**Fig. 3:**
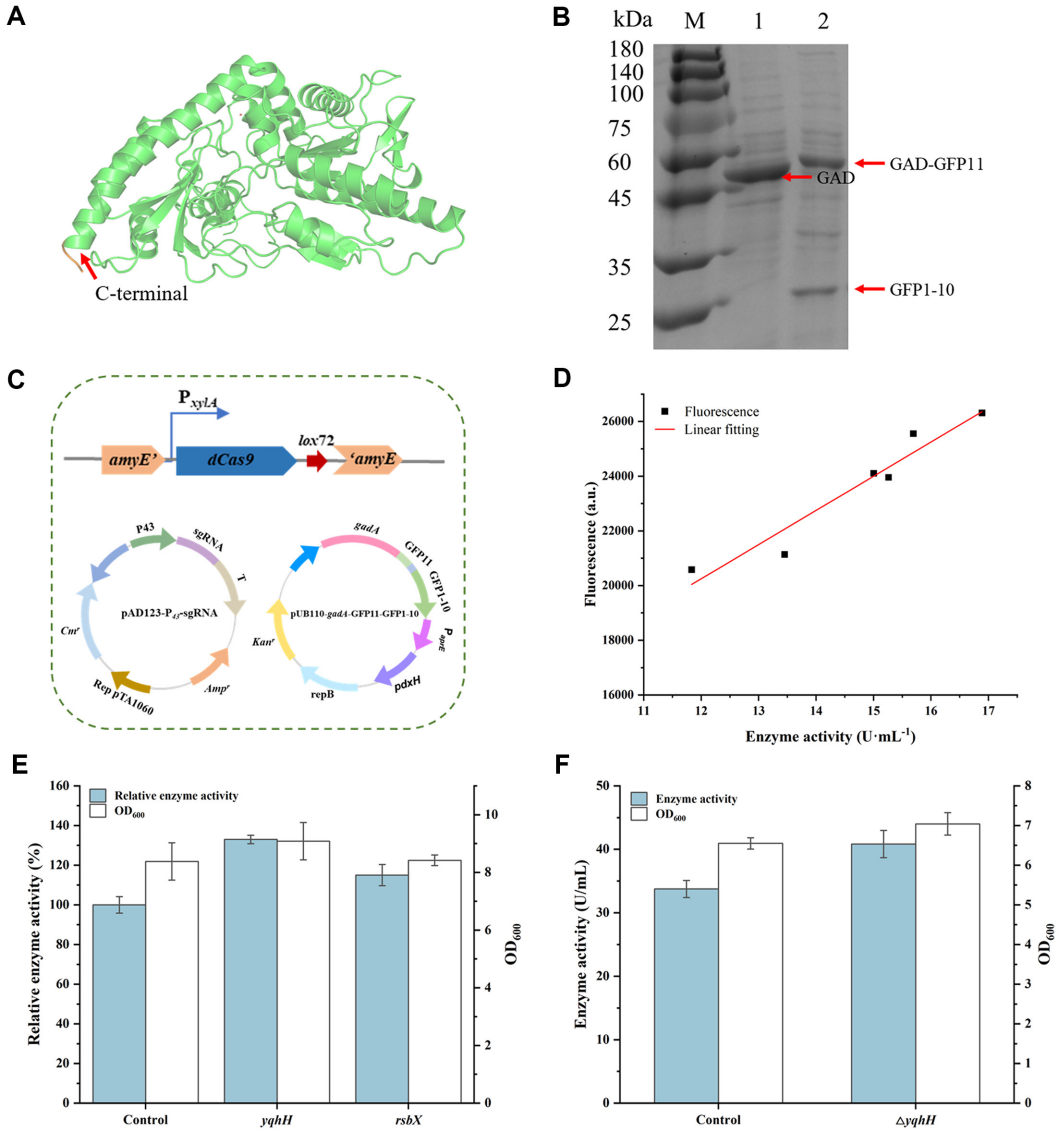
Enhancement of GAD activity via CRISPRi screening technology. (**A**) Structure of GAD. (**B**) SDS-PAGE analysis, M: protein marker; 1: WS9C6D-GAD; 2: WS9Cd-GAD-GFP. (**C**) Genotype schematic of screening library strain. (**D**) Linear correlation between fluorescence and recombinant GAD activity. (**E**) Results of negative factor screening. (**F**) Results of *yqhH* knockout.

GFP has been widely employed as a fluorescent tag. However, the fusion of GFP may impact the expression of the target protein. GFP1-10 and GFP11 are two fragments derived from GFP that do not generate fluorescence independently but can spontaneously bind to form a fluorescent complex [27]. In this study, the small fragment GFP11 was fused to the C-terminal of GAD. As shown in Fig. 3A, the C-terminal of GAD is situated on the exterior of its structure, which helps prevent GFP11 expression from interfering with the catalytic center of GAD. The gene encoding GFP1-10 was located downstream of the GAD-GFP11 fusion and ligated with an RBS sequence, ensuring that their expression levels would be similar when driven by using the same promoter. The GAD and Split-GFP expression plasmid was transformed into the screening strain WS9Cd, which contains *dcas9* expression cassette in its genome, resulting in recombinant strain WS9Cd-GAD-GFP.

After shake-flask fermentation, SDS-PAGE analysis of the intracellular fractions from bacterial samples with an OD_600_ of 5.00 demonstrated that the GAD-GFP11 fusion (54.9 kDa) was expressed normally in strain WS9Cd-GAD-GFP, although its expression level was slightly lower than that of GAD (52.5 kDa) in the control strain WS9C6D-GAD (Fig. 3B).

To investigate whether fluorescence intensity could serve as a screening criterion for strains with higher GAD activity, a mixture of 6,000 sgRNAs was introduced into strain WS9Cd-GAD-GFP (Fig. 3C), and a preliminary small-scale screening was performed. After measuring the fluorescence intensity, strains exhibiting varying fluorescence levels were selected for shake-flask fermentation. Then, following 36 h of cultivation, their fluorescence values and GAD enzyme activities were measured. As shown in Fig. 3D, the fluorescence intensity corresponded well to GAD activity, with linear regression analysis revealing a coefficient of determination (R²) of 0.93, indicating a strong correlation. This suggested that the constructed system was suitable for high-throughput screening.

The sgRNA mixture was introduced into recombinant strain WS9Cd-GAD-GFP to create a CRISPRi library, using strain WS9Cd-GAD-GFP-CK (containing pAD123-P_43_-CK without an N20 sequence on sgRNA) as the control. Using fluorescence intensity to evaluate GAD activity, the CRISPRi library was first screened in 96-well plates, and strains with higher fluorescence values were selected for further analysis in shake-flask fermentation. As shown in Fig. 3E, after 36 h of shake-flask fermentation, the GAD enzyme activities of the two strains were 1.33-and 1.15-fold that of the control strain, respectively. Sequencing analysis revealed that the sgRNAs in these two strains targeted the *yqhH* and *rsbX* genes in the genome, respectively. The *yqhH* gene is expressed during the early stages of forespore formation, and its exact function and regulatory mechanisms require further investigation [28]. The *rsbX* gene encodes a protein serine phosphatase that primarily regulates the activity of the SigB factor, thereby participating in the biofilm formation and general stress response of *B. subtilis*. Under adverse environmental conditions, RsbX interacts with other stress-related proteins, such as RsbT and RsbS, to coordinate the bacterial stress response [29].

To mitigate potential adverse effects of integrating the *dcas9* expression cassette into the genome on the stability of recombinant strain during high-density fermentation, the screened target genes *yqhH* and *rsbX* were selected to be knocked out based on strain WS9C6D to enhance GAD expression. Despite multiple attempts, the *rsbX* gene could not be knocked out, likely due to its critical role in *B. subtilis* biofilm formation and stress response [30]. The *yqhH* gene was successfully knocked out, yielding strain WS9C6DY. After transforming this strain with the promoter optimized plasmid, recombinant strain WS9C6DY-GAD-P_43_ was constructed. As shown in Fig. 3F, after 36 h of shake-flask fermentation, the GAD activity of strain WS9C6DY-GAD-P_43_ reached 40.84 U/ml, which was 1.21-fold that of strain WS9C6D-GAD-P_43_, with no significant differences in bacterial growth observed between the two strains.

### Enhancing GAD Activity through Shake-Flask Fermentation Optimization

To further improve GAD activity, the components of a shake-flask culture medium, including PN concentration, nitrogen sources and carbon sources, were optimized for recombinant strain WS9C6DY-GAD-P_43_.

**Fig. 4:**
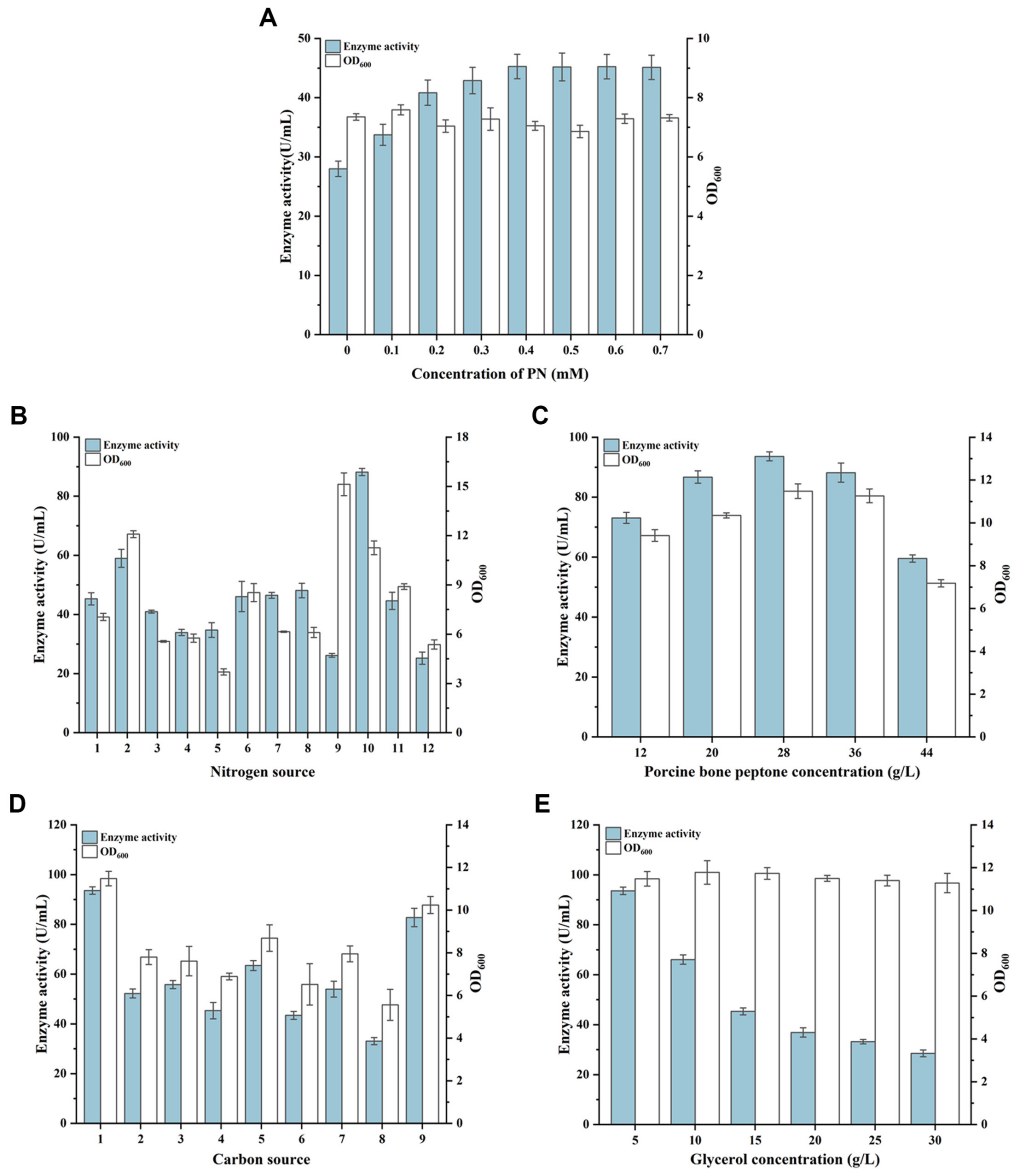
Optimization of shake-flask culture medium. (**A**) Optimization of PN concentration in the original Terrific Broth (**TB**) medium. (**B**) Optimization of nitrogen source types at 0.4 mM PN, 1: TB; 2: soy peptone; 3: industrial peptone; 4: yeast extract; 5: beef extract; 6: industrial soy peptone; 7: industrial yeast powder; 8: tryptone; 9: corn steep liquor powder; 10: porcine bone peptone; 11: fish meal peptone; 12: beef powder. (**C**) Optimization of porcine bone peptone concentration at 0.4 mM PN. (**D**) Optimization of carbon source types at 28 g/l porcine bone peptone and 0.4 mM PN, 1: glycerol; 2: dextrin; 3: maltodextrin; 4: corn dextrin; 5: xylose; 6: fructose; 7: sucrose; 8: glucose; 9: maltose. (**E**) Optimization of glycerol concentration at 28 g/l porcine bone peptone and 0.4 mM PN.

GAD is an enzyme that requires the coenzyme PLP, and optimizing PN addition can regulate PLP synthesis, potentially influencing GAD activity. During shake-flask fermentation, PN was added at concentrations of 0, 0.1, 0.2, 0.3, 0.4, 0.5, 0.6, and 0.7 mM. As shown in Fig. 4A, after 36 h of fermentation, GAD activity increased with higher PN concentrations, achieving the highest activity of 45.28 U/ml at 0.4 mM PN. Further increases in PN concentration did not significantly affect GAD activity, establishing the optimal PN concentration at 0.4 mM.

On the basis of PN concentration optimization, 11 different nitrogen sources were tested individually to replace yeast extract and peptone in TB medium at a concentration of 36 g/l. As shown in Fig. 4B, after 36 h of shake-flask fermentation, the highest GAD activity of 88.19 U/ml was achieved using porcine bone peptone as the nitrogen source. The optimal concentration of the porcine bone peptone was then investigated at 12, 20, 28, 36, and 44 g/l. As shown in Fig. 4C, after 36 h of shake-flask fermentation, the highest GAD activity of 93.61 U/ml was achieved at a concentration of 28 g/l, which was used in subsequent studies.

Following nitrogen source optimization, 8 different carbon sources were individually tested to replace glycerol in the medium at a concentration of 5 g/l. As shown in Fig. 4D, after 36 h of shake-flask fermentation, glycerol yielded the highest GAD activity of 93.61 U/ml. The optimal glycerol concentration was then investigated at 5, 10, 15, 20, 25, and 30 g/l. As shown in Fig. 4E, after 36 h of shake-flask fermentation, the highest GAD activity was achieved at a concentration of 5 g/l, which was used in subsequent studies.

### High-Density Fermentation of Recombinant Strain in a 3-L Fermenter

**Fig. 5:**
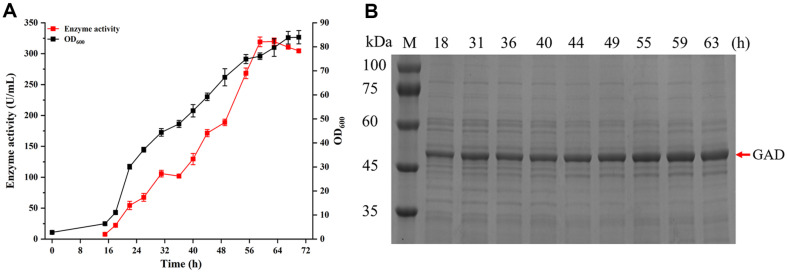
Fermentation of the recombinant strain WS9C6DY-GAD-P_43_ in a 3-L fermenter. (**A**) OD_600_ and enzyme activity. (**B**) SDS-PAGE analysis.

Based on the results of shake-flask optimization, the medium for the 3-L fermenter utilized porcine bone peptone as the nitrogen source and glycerol as the carbon source. PN was added initially and supplemented every 24 h. The cultivation of recombinant strain WS9C6DY-GAD-P_43_ was carried out at 37°C, pH 6.8, and 30%dissolved oxygen. As shown in Fig. 5A, GAD activity peaked at 319.62 U/ml after 63 h of fermentation, which was 3.41-fold of GAD activity observed in shake-flask fermentation, representing the highest food-grade activity of GAD reported to date. SDS-PAGE analysis of the intracellular samples at OD_600_ 5.00 collected at various fermentation times revealed distinct bands near the theoretical size of GAD (52.5 kDa), which increased with fermentation time, corresponding to the trend in enzyme activity (Fig. 5B).

### Enzymatic Properties of Recombinant GAD

After enhancing GAD activity in *B. subtilis*, we investigated the enzymatic properties of recombinant GAD, including the kinetic parameters, optimal temperature, thermal stability, optimal pH, and pH stability.

**Fig. 6:**
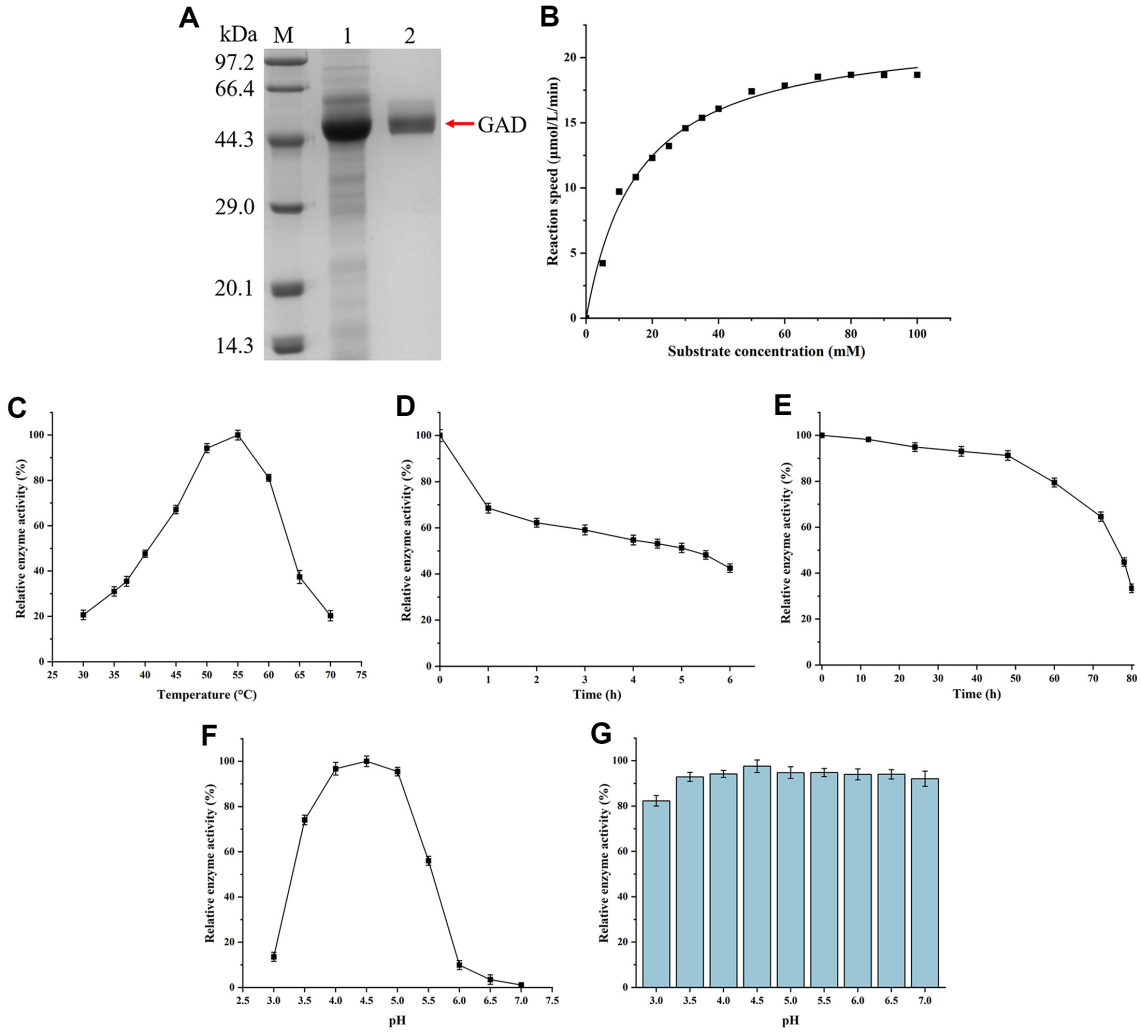
Enzymatic properties of recombinant GAD. (**A**) SDS-PAGE analysis, M: protein marker; 1: cell lysate; 2: purified enzyme. (**B**) Kinetic analysis of GAD at different MSG concentrations (0-100 mM). (**C**) Optimal temperature analysis within the range of 55-70°C under pH 4.5. (**D**) Thermal stability analysis at 55°C under pH 4.5. (**E**) Thermal stability analysis at 37°C under pH 4.5. (**F**) Optimal pH analysis under 37°C. (**G**) Stability analysis within the pH range of 3.0-7.0 under 4°C.

The recombinant GAD was purified through cation-exchange chromatography. As shown in Fig. 6A, the purified GAD appeared as a single band in SDS-PAGE analysis, consistent with the theoretical molecular weight of 52.5 kDa. The specific activity of GAD was measured to be 30.41 U/mg. By measuring GAD activity at different MSG concentrations (0-100 mM) and fitting the data using Origin software (Fig. 6B), the kinetic parameters of V_max_, *K*_m_ and *k*_cat_ were calculated to be 22.27 μmol/l/min, 15.77 mM and 36.26/s, respectively.

Temperature is a crucial factor affecting the catalytic reaction rate of enzyme, with both low and high temperatures adversely impacting enzyme activity. To determine the optimal temperature for recombinant GAD, enzyme activity was measured at temperatures ranging from 30 to 70°C. As shown in Fig. 6C, enzyme activity gradually increased with temperature from 30 to 55°C, and then decreased from 55 to 70°C, indicating an optimal temperature of 55°C. The thermal stability of GAD was evaluated at both 55 and 37°C. As shown in Fig. 6D-6E, the half-life of GAD at 55 and 37°C was 5.2 and 76 h, respectively, demonstrating a significant improvement in GAD stability at lower temperature.

Similar to temperature, pH is another critical factor influencing enzyme activity. To ascertain the optimal pH for GAD, enzyme activity was measured across a pH range of 3.0 to7.0. As shown in Fig. 6F, enzyme activity gradually increased from pH 3.0 to 4.5, reaching an optimal pH of 4.5, and then gradually decreasing from pH 4.5 to 7.0. The enzyme retained over 95% activity within the pH range of 4.0 to 5.0, but was nearly inactivated at pH levels above 6.0. Additionally, the stability of GAD was assessed at pH 3.0 to 7.0 by placing the enzyme solution at the corresponding pH levels at 4°C for 24 h, followed by measuring their enzyme activity. As shown in Fig. 6G, relative enzyme activity remained above 90% within the pH range of 3.5 to 7.0, indicating that recombinant GAD exhibits good pH stability.

### Process Optimization of GABA Preparation from MSG

Enzymatic catalysis can utilize either free enzymes or recombinant bacterial cells. The latter eliminates the need for enzyme separation and extraction, reducing preparation costs and enhancing enzyme stability in a cellular environment [8, 31]. Therefore, GAD-recombinant bacterial cells were used as catalysts for GABA preparation, and reaction conditions were optimized for high-level GABA production.

**Fig. 7:**
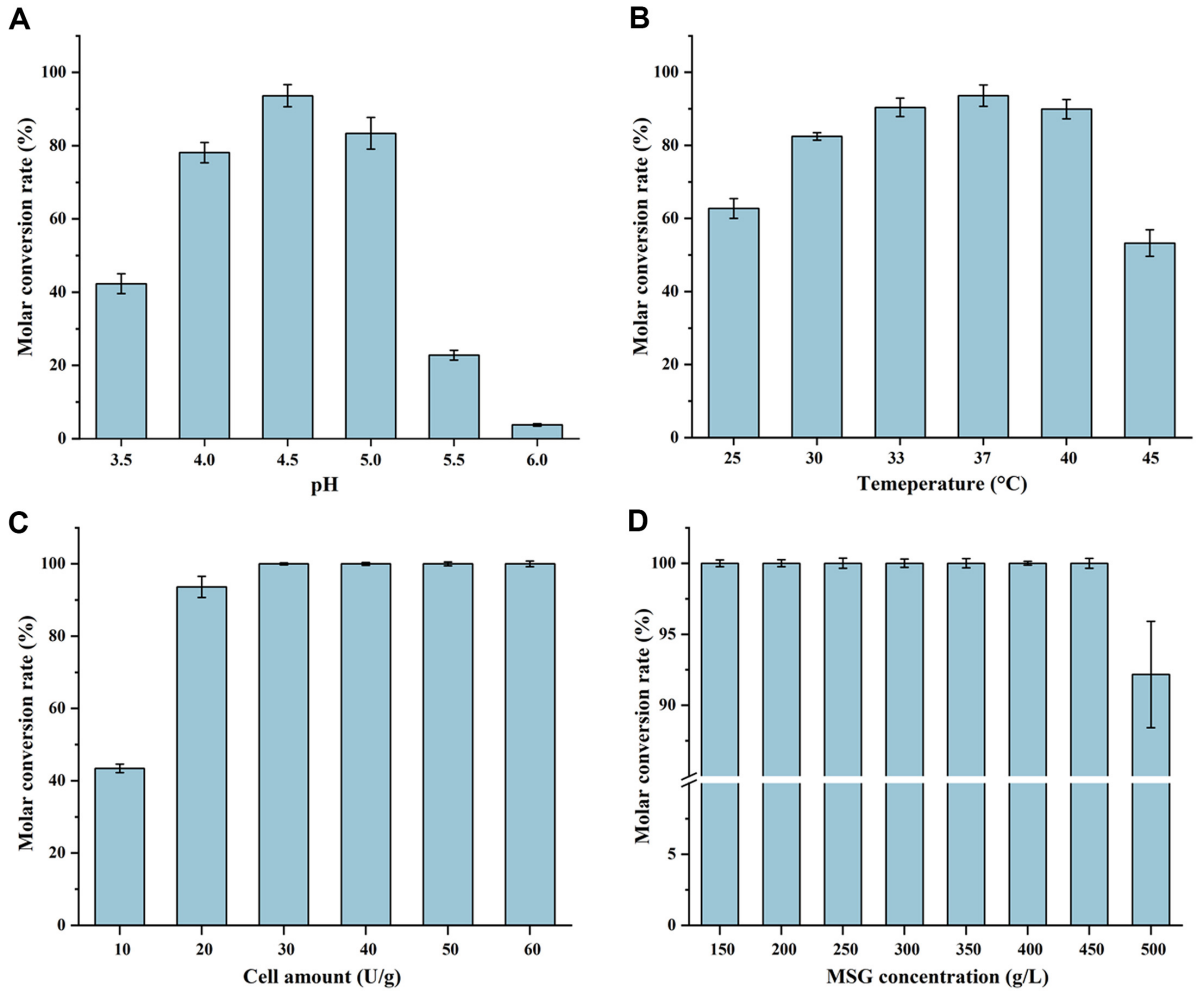
Preparation of GABA from MSG. (**A**) Optimization of pH under 150 g/l MSG at 37°C with GAD-recombinant bacterial cells added at 20 U/g MSG. (**B**) Optimization of temperature under 150 g/l MSG, pH 4.5 with GAD-recombinant bacterial cells added at 20 U/g MSG. (**C**) Optimization of cell concentration under 150 g/l MSG, pH 4.5 at 37°C. (**D**) Optimization of MSG concentration under pH 4.5 at 37°C with GAD-recombinant bacterial cells added at 30 U/g MSG.

For pH optimization, a 150 g/l MSG solution was prepared with initial pH values of 3.5, 4.0, 4.5, 5.0, 5.5, and 6.0. The GAD-recombinant bacterial cells were added at a concentration of 20 U/g MSG, and the catalytic reaction was performed in a water bath shaker at 37°C and 200 rpm for 8 h. The conversion of MSG to GABA consumed H+ ions, resulting in a gradual increase in pH, which was adjusted using 6 mM H_2_SO_4_ every 1.5 h. The molar conversion rate of MSG to GABA was calculated after the reaction. As shown in Fig. 7A, the optimum pH for GABA preparation was found to be 4.5, yielding the highest molar conversion rate of 93.64%, which was used in subsequent studies.

For temperature optimization, GABA preparation using whole-cell catalysis was performed at temperatures of 25, 30, 33, 37, 40, and 45°C. The molar conversion rate of MSG to GABA was calculated after the reaction. As shown in Fig. 7B, the molar conversion rate increased with temperature, reaching a maximum of 93.64% at 37°C, likely due to the high catalytic activity and excellent stability of GAD at this temperature. This temperature was also used in later studies.

For cell amount optimization, different cell concentrations of 10, 20, 30, 40, 50, and 60 U/g substrate were tested with the 150 g/l MSG solution. The molar conversion rate of MSG to GABA was calculated after the reaction. As shown in Fig. 7C, when the cell concentration was less than 30 U/g MSG, the conversion rate increased with the cell amount, reaching a maximum of 100% at 30 U/g substrate. To avoid increasing preparation costs with excessive cell amounts, 30 U/g MSG was selected for further studies.

For substrate concentration optimization, the initial MSG concentration was set at 150 g/l for a 4 h reaction. The MSG concentration was then either maintained or increased to 200, 250, 300, 350, 400, 450, and 500 g/l by adding 50 g/l every 2 h until the desired concentrations were reached, with a total reaction time of 24 h. The molar conversion rate of MSG to GABA was calculated afterward. As shown in Fig. 7D, the molar conversion rate remained at 100% for substrate concentrations up to 450 g/l but decreased to 92.17% at 500 g/l. To maximize substrate utilization and GAD-recombinant bacterial efficiency, 450 g/l was selected as the optimal MSG concentration, with a final cell amount of 13.5 U/ml.

### Process Optimization of GABA Preparation from L-Glu

In the previous section, MSG was used as the substrate for GABA preparation due to its higher solubility and lower cost compared to L-Glu. However, the high MSG concentration required substantial amounts of H_2_SO_4_ for pH adjustment, increasing preparation cost and complexity. MSG aqueous solutions have been shown to be nearly neutral, whereas L-Glu aqueous solutions are acidic, with a saturated aqueous solution pH of about 3.2, which helps maintain an acidic reaction environment. Therefore, GABA preparation from L-Glu was also investigated.

**Fig. 8:**
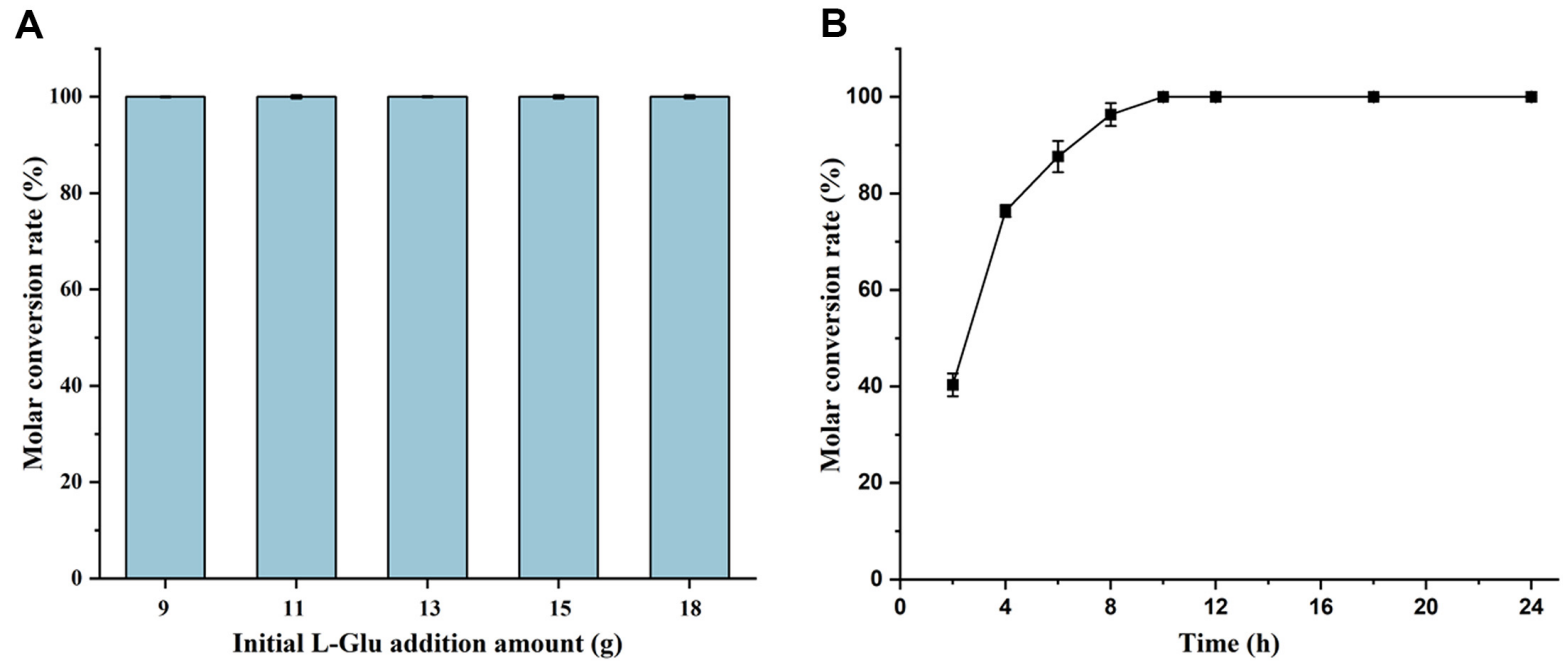
Preparation of GABA from L-Glu. (**A**) Optimization of L-Glu concentration under pH 4.5 at 37°C with GADrecombinant bacterial cells added at 13.5 U/ml. (**B**) Optimization of reaction time with an initial L-Glu addition of 18 g.

Due to the low solubility, 9, 11, 13, 15, and 18 g L-Glu were added directly to a 20 ml reaction system, resulting in an incompletely dissolved saturation state to achieve high substrate concentrations. During the catalysis, dissolved L-Glu was converted to GABA while solid L-Glu dissolved slowly. Based on the results from MSG, the reaction was conducted at pH 4.5, 37°C, and 200 rpm for 24 h. For this reaction, GAD-recombinant bacterial cells were added at a final concentration of 13.5 U/ml, consistent with the preparation from 450 g/l MSG. The molar conversion rate of L-Glu to GABA was calculated after the reaction. As shown in Fig. 8A, the molar conversion rate reached 100% for all five different substrate concentrations, indicating high enzymatic conversion efficiency for GABA preparation from L-Glu.

Subsequently, the conversion rate for the sample with 18 g of initial L-Glu addition was analyzed at different reaction times. As shown in Fig. 8B, the molar conversion rate reached 100% at 10 h and remained unchanged thereafter. At this point, the GABA production was 481.62 g/l, yielding 48.16 g/l/h, which was the highest yield of GABA reported to date.

### Effect of GABA Addition on Gut Microbiota in *In Vitro* Fermentation

Gut microbiota is a crucial component of the human digestive ecosystem and comprises a vast majority of the human microbiota population [32]. These microorganisms utilize gut nutrients through various metabolic pathways and play significant roles in human growth and development, energy metabolism, immune function, and nervous system regulation. GABA, an important neurotransmitter, is commonly used as a dietary supplement, probiotic food ingredient, and medication. In this study we investigated the effect of GABA addition on gut microbiota through an *in vitro* fermentation method.

**Fig. 9:**
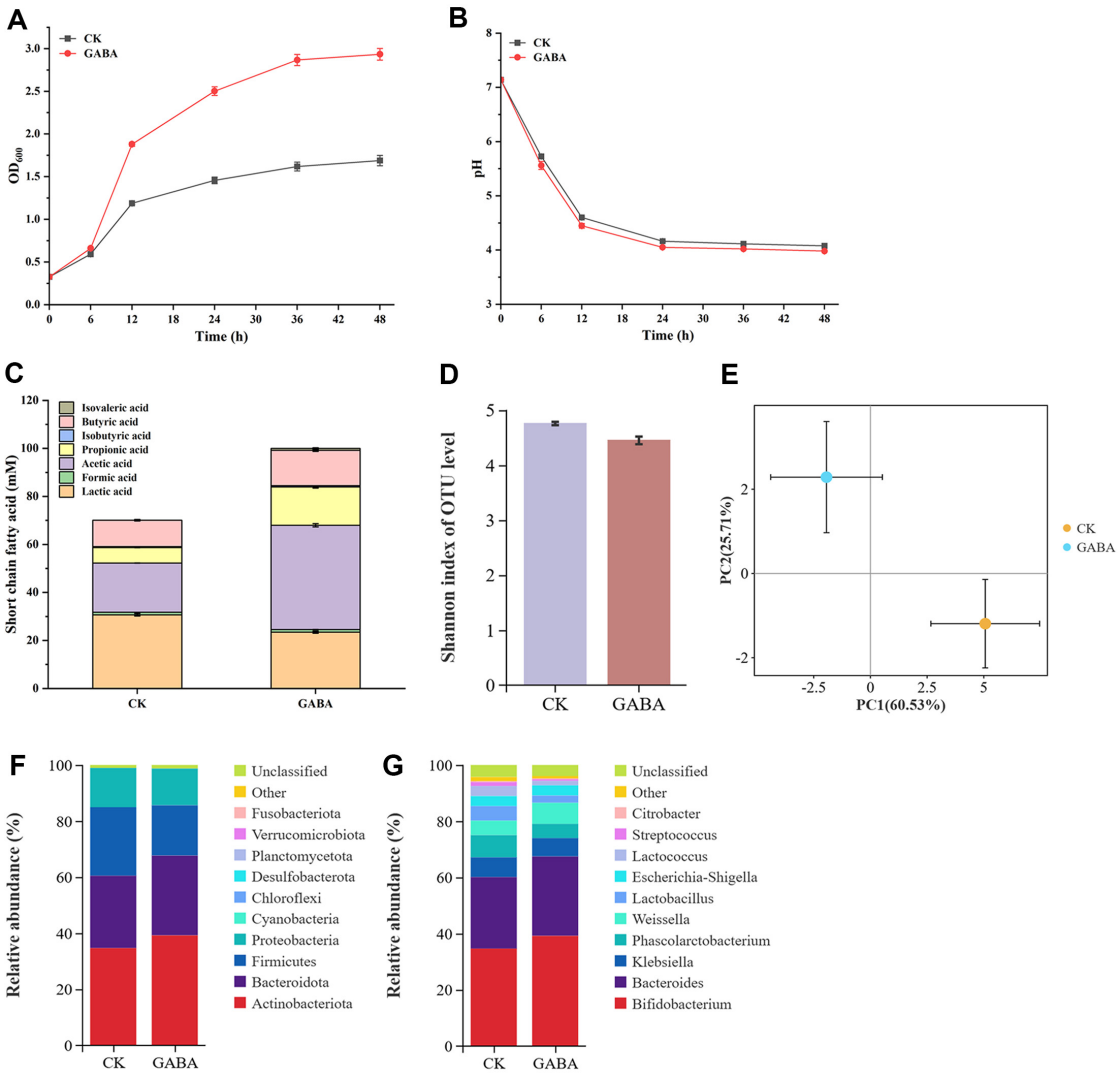
Effects of GABA addition on gut microbiota. (**A**) Cell growth. (**B**) Culture pH value. (**C**) Production of shortchain fatty acids. (**D**) Alpha-diversity analysis. (**E**) Principal coordinate analysis (PCoA). (**F**) Abundance of gut microbiota at phylum level. (**G**) Abundance of gut microbiota at genus level.

Using human fecal mixed bacteria cultured without GABA as the control, the experimental group was cultured with an addition of 5 g/l GABA. As shown in Fig. 9A-9C, compared to the control, the addition of GABA resulted in improved microbial cell growth, indicating that GABA can be effectively utilized by intestinal microorganisms, with no significant difference in pH values observed. After utilizing external nutrients, intestinal microorganisms can produce short-chain fatty acids [33]. The results indicated that GABA addition significantly increased the productions of acetic acid, butyric acid, and propionic acid, while decreasing lactic acid production. Notably, acetic acid production was the highest, reaching 1.3-fold that of the control. Acetic acid can be absorbed by intestinal epithelial cells, serving as an energy source for the body and playing physiological roles in preventing intestinal inflammation.

The Shannon index and PCoA were used to investigate the α- and β-diversity of the two groups at OTUs level, respectively. As shown in Fig. 9D-9E, the experimental group exhibited a minor decrease in the Shannon index compared to the control, suggesting that GABA addition reduced the α-diversity of gut microbiota. PCoA revealed contribution values of PC1 and PC2 at 60.53% and 25.71%, respectively, indicating significant microbial community separation between the two groups and demonstrating that GABA addition significantly affected gut microbiota community distribution.

The effect of GABA addition on the abundance of gut microbiota was analyzed at both phylum and genus levels. As shown in Fig. 9F-9G, at the phylum level, GABA addition increased the relative abundances of *Actinobacteriota* and *Bacteroidota* by 13.04% and 10.28%, respectively. *Actinobacteriota* are gram-positive bacteria that play an important role in the decomposition of organic material, and *Bacteroidota* are gram-negative anaerobic bacteria that produce anaerobic respiration products, such as acetic acid, succinic acid, and isovaleric acid, consistent with the observed changes in short-chain fatty acid content. At the genus level, the relative abundances of *Bifidobacterium* and *Bacteroides* increased by 13.07% and 11.00%, respectively. *Bifidobacterium*, a beneficial bacterial of *Actinobacteriota*, plays vital physiological roles in the gut and its reduced content is associated with obesity, diabetes, allergies, and other diseases [34]. *Bacteroides*, a neutral bacterial of *Bacteroidota*, is important for protein metabolism and the decomposition of non-fibrous carbohydrates [35].

In summary, this study enhanced GAD activity in *B. subtilis* for the first time by increasing coenzyme PLP synthesis, modifying the host strain using CRISPRi screening technology, and optimizing fermentation culture conditions. The GAD activity of the recombinant strain WS9C6DY-GAD-P_43_ reached 319.62 U/ml in a 3-L fermenter without antibiotic selection pressure, marking the highest food-grade activity reported to date. Following this, based on the investigation of the enzymatic properties of recombinant GAD, GABA preparation conditions for the whole-cell catalysis system were optimized using MSG and L-Glu as substrates, yielding the highest GABA production with a 100% molar conversion rate of 274.40 and 481.62 g/l, respectively. The latter had a yield of 48.16 g/l/h, the highest reported thus far. Finally, the effects of GABA addition on gut microbiota were examined, revealing that GABA addition promoted microbial cell growth, affected gut microbiota diversity, and increased the relative abundances of *Actinobacteriota* and *Bacteroidota* by 13.04% and 10.28%, respectively. In this study, the highest-ever food-grade expression of GAD and GABA preparation was achieved, thereby promoting the industrial production and application of GABA.

## Supplemental Materials

Supplementary data for this paper are available on-line only at http://jmb.or.kr.



## Figures and Tables

**Table 1:** Promoters used in this study.

Promoters	Origins	Properties	Expressed proteins
P*_HpaII_*-P*_amyQ'_*	*Staphylococcus aureus* (P*_HpaII_*), *B. subtilis* (P*_amyQ'_*) Strong constitutive promoter constructed with promoters of plasmid recombination enzyme (P*_HpaII_*) and α-amylase (P*_amyQ'_*) [36]	Cyclodextrin glycosyltransferase, pullulanase
P*_spoVG_*	*B. subtilis*	Promoter of RNA-binding regulatory protein SpoVG, constitutively expressed at stationary phase [37]	Keratinase
P*_ahpF_*	*B. subtilis*	Promoter of NADH dehydrogenase, constitutively expressed at stationary phase [38]	None
P*_tufA_*	*B. subtilis*	Promoter of elongation factor Tu, constitutively expressed [39]	Pullulanase
P*_ylb'_*	*B. subtilis*	Promoter of putative N-acetyltransferase YlbP, constitutively expressed at late log phase and stationary phase [40]	Pullulanase, organophosphorus hydrolase, EGFP, RFP
P*_aprE_*	*B. subtilis*	Promoter of alkaline protease, constitutively expressed [36]	Cyclodextrin glycosyltransferase
P_43_	*B. subtilis*	Strong constitutive promoter constructed with promoters of levansucrase and cytidine deaminase [25]	Cytidine dehydrogenase, methyl parathion hydrolase, EGFP, RFP
P*_nprE_*	*B. subtilis*	Promoter of neutral protease, constitutively expressed [36]	Cyclodextrin glycosyltransferase
P*_fusA_*	*B. subtilis*	Promoter of elongation factor G, constitutively expressed [39]	Pullulanase
