# Supplementary material for: Enhancing High-Level Food-Grade Expression of Glutamate Decarboxylase and Its Application in the Production of γ-Aminobutyric Acid
Source: J Microbiol Biotechnol. 2024 Dec 12;35:e2410013. doi: 10.4014/jmb.2410.10013 (PMC11813360; doi:10.4014/jmb.2410.10013)
Supplement: Supplementary file 1 [file jmb-35-e2410013-supple.pdf]

J. Microbiol. Biotechnol. 2025. 35: e2410013
https://doi.org/10.4014/jmb.2410.10013

Supplemental tables

Enhancing High-Level Food-Grade Expression of Glutamate Decarboxylase and Its Application in the Production of γ-Aminobutyric Acid

Kang Zhang[1,2,3†], Huihui Lv[1,2,3†], Xinrui Yu[1,2,3], Xuyang Zhu[1,2,3], Sheng Chen[1,2,3], Jing Wu[1,2,3*]

[1]School of Biotechnology and Key Laboratory of Industrial Biotechnology Ministry of Education, Jiangnan University, 1800 Lihu Avenue, Wuxi, 214122, China
[2]State Key Laboratory of Food Science and Resources, Jiangnan University, 1800 Lihu Avenue, Wuxi, 214122, China
[3]International Joint Laboratory on Food Safety, Jiangnan University, 1800 Lihu Avenue, Wuxi, 214122, China

[†]Both authors contributed equally to this work

*Corresponding author:

Jing Wu

-mail: jingwu@jiangnan.edu.cn

Table S1. Strains and plasmids used in this study

Strains/Plasmids	Characteristics	Resource
Strains		
B. subtilis SCK6D	*B. subtilis* SCK6, Δ*dal*	Lab stock
WS9C6D	*B. subtilis* WS9, *amyE*::*comK*, *nprE*::*gadA*, *aprE*::*gadA*, *nprB*::*gadA*, *srfA*::*gadA*, *mpr*::*gadA*, *bpr*::*gadA*, Δ*dal*::*gadA*	Lab stock
WS9C6D-GAD	WS9C6D harboring pUBDAL-*gadA*	Lab stock
WS9C6D-P$_{ahpF}$-GAD	WS9C6D harboring pUBDAL-P$_{ahpF}$-*gadA*	This study
WS9C6D-P$_{spoVG}$-GAD	WS9C6D harboring pUBDAL-P$_{spoVG}$-*gadA*	This study
WS9C6D-P$_{tufA}$-GAD	WS9C6D harboring pUBDAL-P$_{tufA}$-*gadA*	This study
WS9C6D-P$_{ylb'}$-GAD	WS9C6D harboring pUBDAL-P$_{ylb'}$-*gadA*	This study
WS9C6D-GAD-P$_{43}$	WS9C6D harboring pUBDAL-*gadA*-P$_{43}$	This study
WS9C6D-GAD-P$_{spoVG}$	WS9C6D harboring pUBDAL-*gadA*-P$_{spoVG}$	This study
WS9C6D-GAD-P$_{tufA}$	WS9C6D harboring pUBDAL-*gadA*-P$_{tufA}$	This study
WS9C6D-GAD-P$_{nprE}$	WS9C6D harboring pUBDAL-*gadA*-P$_{nprE}$	This study
WS9C6D-GAD-P$_{ahpF}$	WS9C6D harboring pUBDAL-*gadA*-P$_{ahpF}$	This study
WS9C6D-GAD-P$_{fusA}$	WS9C6D harboring pUBDAL-*gadA*-P$_{fusA}$	This study
WS9Cd	*B. subtilis* WS9, *amyE*::*comK*, *dCas9*	Lab stock
WS9Cd-GAD-GFP	WS9C6D harboring pUB110-*gadA*-GFP11-GFP1-10	This study
WS9Cd-GAD-GFP-CK	WS9C6D harboring pUB110-*gadA*-GFP11-GFP1-10, pAD123-P$_{43}$-CK	This study
WS9C6DY	WS9C6D, Δ*yqhH*	This study
WS9C6DY-GAD-P$_{43}$	WS9C6DY harboring pUBDAL-*gadA*-P$_{43}$	This study
Plasmids		
pUBDAL-*gadA*	P$_{HpaII}$-P$_{amyQ'}$-*gadA*, P$_{aprE}$-*pdxH*, RBS1-*dal*	Lab stock
pE194-Cre	pDG148-Cre, PE194 temperature-sensitive replicon	Lab stock
pHY300PLK	*tet*^{r}, *amp*^{r}, *E. coli* and *B. subtilis* shuttle vector	Lab stock
pUBDAL-P$_{spoVG}$-*gadA*	P$_{spoVG}$-*gadA*, P$_{aprE}$-*pdxH*, RBS1-*dal*	This study
pUBDAL-P$_{ahpF}$-*gadA*	P$_{ahpF}$-*gadA*, P$_{aprE}$-*pdxH*, RBS1-*dal*	This study
pUBDAL-P$_{tufA}$-*gadA*	P$_{tufA}$-*gadA*, P$_{aprE}$-*pdxH*, RBS1-*dal*	This study

pUBDAL-P$_{ylb'}$-*gadA*	P$_{ylb'}$-*gadA*, P$_{aprE}$-*pdxH*, RBS1-*dal*	This study
pUBDAL-*gadA*-P$_{43}$	P$_{HpaII}$-P$_{amyQ'}$-*gadA*, P$_{43}$-*pdxH*, RBS1-*dal*	This study
pUBDAL-*gadA*-P$_{spoVG}$	P$_{HpaII}$-P$_{amyQ'}$-*gadA*, P$_{spoVG}$-*pdxH*, RBS1-*dal*	This study
pUBDAL-*gadA*-P$_{tufA}$	P$_{HpaII}$-P$_{amyQ'}$-*gadA*, P$_{tufA}$-*pdxH*, RBS1-*dal*	This study
pUBDAL-*gadA*-P$_{nprE}$	P$_{HpaII}$-P$_{amyQ'}$-*gadA*, P$_{nprE}$-*pdxH*, RBS1-*dal*	This study
pUBDAL-*gadA*-P$_{ahpF}$	P$_{HpaII}$-P$_{amyQ'}$-*gadA*, P$_{ahpF}$-*pdxH*, RBS1-*dal*	This study
pUBDAL-*gadA*-P$_{fusA}$	P$_{HpaII}$-P$_{amyQ'}$-*gadA*, P$_{fusA}$-*pdxH*, RBS1-*dal*	This study
pAD123-P$_{43}$-CK	cm^r, P$_{43}$-sgRNA, no N20 sequence on sgRNA	Lab stock
pAD123-P$_{43}$-sgRNA	cm^r, P$_{43}$-sgRNA, sgRNA mixture	Lab stock
pUB110-GFP11-GFP1-10	kan^r, P$_{amyQ'}$-β-Gal-GFP11-GFP1-10	Lab stock
pUB110-*gadA*-GFP11-GFP1-10	kan^r, P$_{HpaII}$-P$_{amyQ'}$-*gadA*-GFP11-GFP1-10, P$_{ahpF}$-*pdxH*	This study

Table S2. Primers used in this study.

Primers	Sequence 5′-3′
P1	GTTTAAAGGTGGAGATTTTTTGAGTGA
P2	ATGGACCAGAAGCTGTTAACG
P3	TGGAGATTTTTTGAGTGATCCAAAAGCAGTCCACACAAAACATG
P4	GTTAACAGCTTCTGGTCCATAGTAGTTCACCACCTTTTCCC
P5	TGGAGATTTTTTGAGTGATCCCTGTATTAAAAACACGGTCAGTTTC
P6	GTTAACAGCTTCTGGTCCATAATGTATATTCCTCCTAAAAATGTATTAGAAAG C
P7	TGGAGATTTTTTGAGTGATCTTGATTTTGCCGCTTAACTCAAG
P8	GTTAACAGCTTCTGGTCCATTCTAAAATCCTCCTTAAGAGCTTTTAATTAG
P9	TGGAGATTTTTTGAGTGATCACTTCTCAAAGATCCCATTTAAAAATTTT
P10	GTTAACAGCTTCTGGTCCATACGTTCTACCTTTGTCAAACAAATC
P11	ATGAGCGATAATGACGAGCTG
P12	CCGTCTGTACGTTCCTAAACTAGT
P13	CTGTACGTTCCTAAACTAGTATTTTACATTTTTAGAAATGGGCGTG
P14	AGCTCGTCATTATCGCTCATTTATATTTACATAATCGCGCGC
P15	CTGTACGTTCCTAAACTAGTCAAAAGCAGTCCACACAAAACATG
P16	AGCTCGTCATTATCGCTCATAGTAGTTCACCACCTTTTCCC
P17	CTGTACGTTCCTAAACTAGTTTGATTTTGCCGCTTAACTCAAG
P18	GTCATTATCGCTCATTCTAAAATCCTCCTTAAGAGCTTTTAATTAG
P19	CTGTACGTTCCTAAACTAGTCACCCGCCAAGAACATTGTG
P20	GTCATTATCGCTCATAATAAATCCCCCTTTTTGAAAATACTG
P21	CTGTACGTTCCTAAACTAGTCCTGTATTAAAAACACGGTCAGTTTC
P22	GTCATTATCGCTCATAATGTATATTCCTCCTAAAAATGTATTAGAAAGC
P23	CTGTACGTTCCTAAACTAGTCTGGTGCTGCTGTTAAGAAAC
P24	AGCTCGTCATTATCGCTCATTGGGTAATTTCTCCTTCCTTATTAGG
P25	AGAACAGCTTTAAACACACCGGCGGAGGCTCTGGC
P26	TTTTTATTACCAAGCTTTTATTTTTCATTCGGATCTTTAGACAGAACTG
P27	CTAAAGATCCGAATGAAAAATAAAAGCTTGGTAATAAAAAAACACCTCC
P28	CCTCCGCCAGAGCCTCCGCCGGTGTGTTTAAAGCTGTTCTGCTG
P29	CCAAATATGCATAGCATCATCACTGC
P30	CTTCGTATAATGTATGCTATACGAACGGTATTGCTGTTGCCCAGCATATAGTG
P31	CTTCGTATAGCATACATTATACGAACGGTAATGAATACAGAAATGATCTACGA

	TGC
P32	TTCAGCGTCATATATACCGCTTCTC
P33	TTCGTATAGCATACATTATACGAAGTTATTTCAACAAACGGGCCATATTGTTG
P34	TTCGTATAATGTATGCTATACGAAGTTATAAGCTTCTAGAGATCTGCAGGTC
P35	CCTACGGGGCGCAG
P36	GGACTACHVGGTATCTAAT
